# Supplementary material for: Novel near-infrared emission from crystal defects in MoS2 multilayer flakes
Source: Nat Commun. 2016 Oct 4;7:13044. doi: 10.1038/ncomms13044 (PMC5059461; doi:10.1038/ncomms13044)
Supplement: Supplementary Information — Supplementary Figures 1-14, Supplementary Notes 1-3 and Supplementary References [file ncomms13044-s1.pdf]

Supplementary Figure 1: Gaussian deconvolution of the CL spectrum of MoS_2 flake, shown in Figure 1

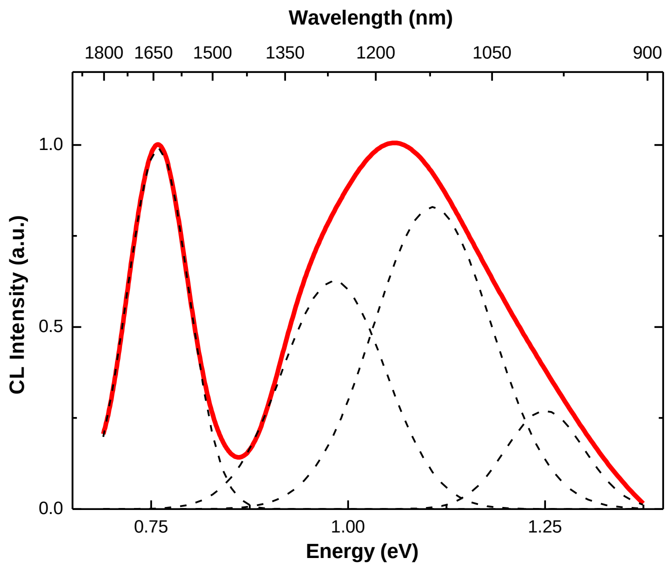

It is worth noticing the onset of a narrow emission peaked at 0.76 eV not present in pristine Molybdenite

Supplementary Figure 2 Gaussian deconvolution of the CL spectrum of pristine Molybdenite, shown in Figure 1.

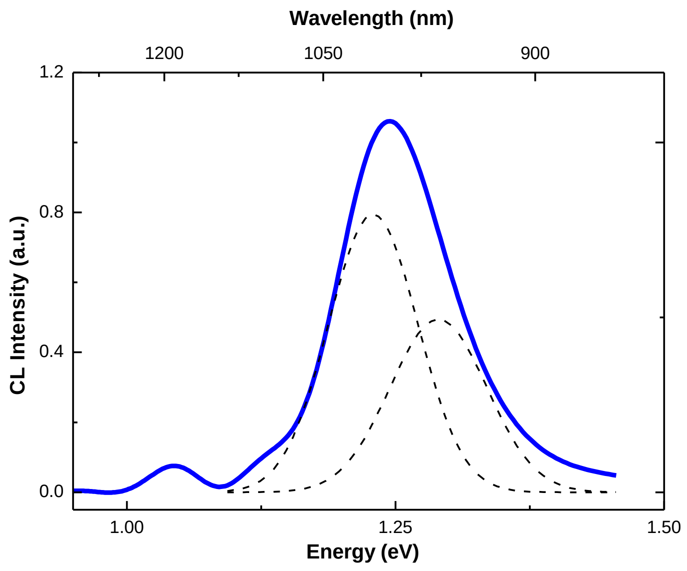

Gaussian deconvolution of the CL peak of pristine Molybdenite in Figure 1. The band-to-band transition of the bulk MoS_2[1] at 1.29 eV is clearly shown.

Supplementary Figure 3: Cathodoluminescence spectroscopy of pristine and cracked Molybdenite

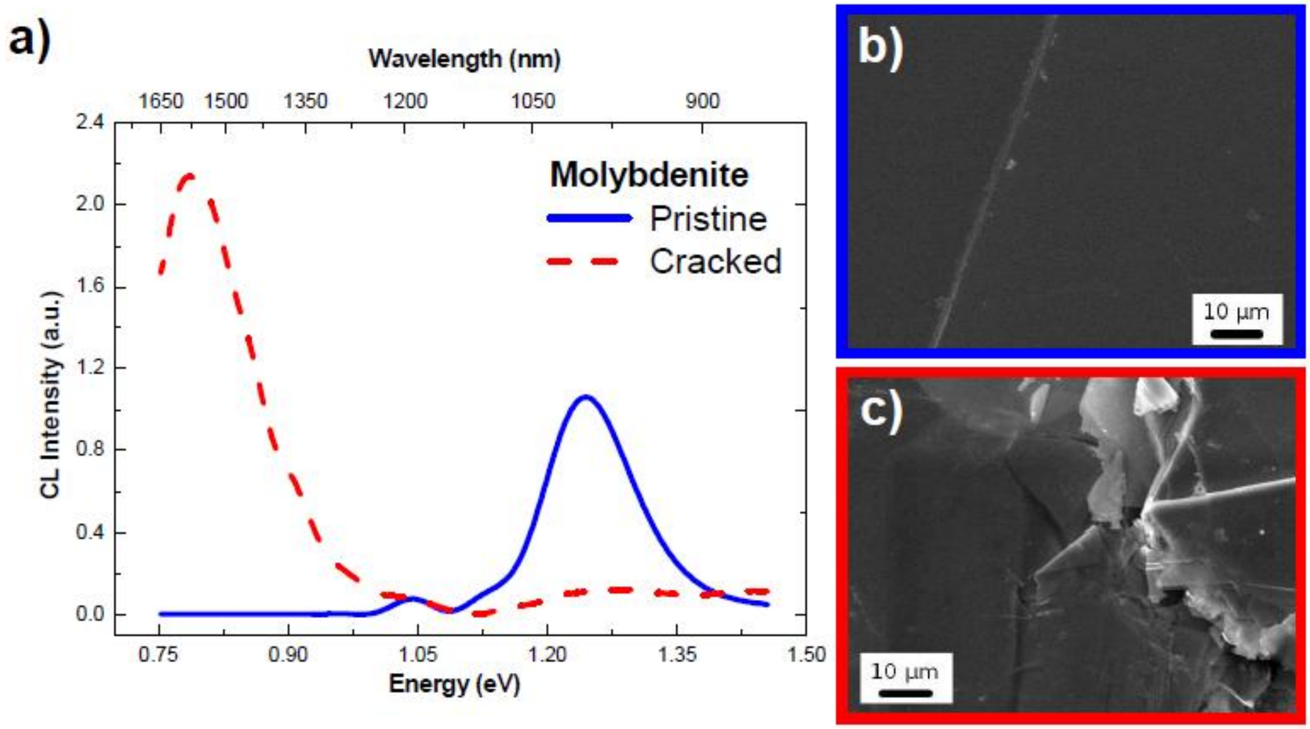

a) CL spectra of the pristine (blue full line) and cracked (red dashed line) Molybdenite. b) and c) SE images of the Molybdenite surfaces where the CL spectra are taken. The colored frames are used as color guidelines

Supplementary Figure 4: Comparison between CL spectra before and after electron beam irradiation

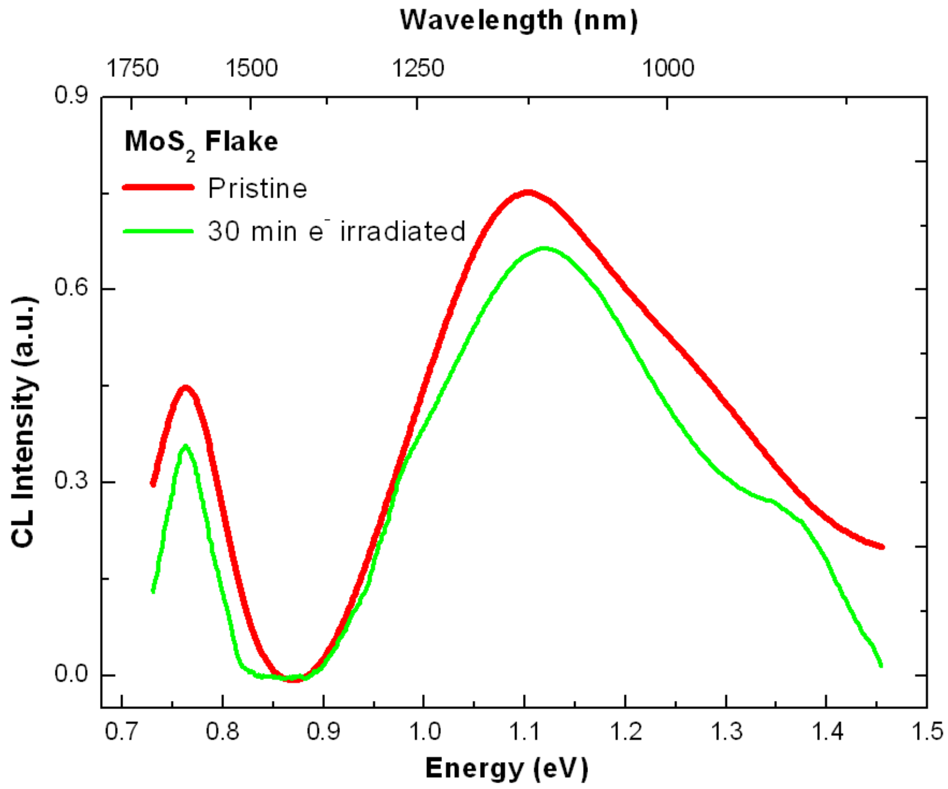

CL spectra of MoS2 flake before (red line) and after 30 minutes electron beam irradiation (green line).

Supplementary Figure 5: Scanning transmission electron microscopy analysis of ripplocations

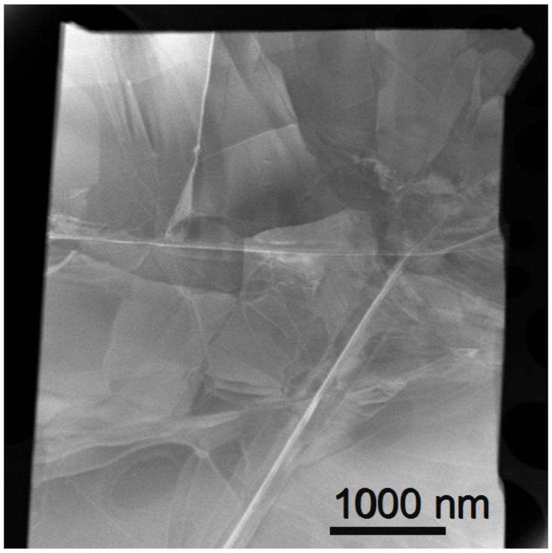

STEM micrograph enlargement of one portion of the flake shown in Figure 1a showing the homogeneous distribution of ripplocations all across the flake.

Supplementary Figure 6: Z-contrast analysis and intensity of a typical flake edge

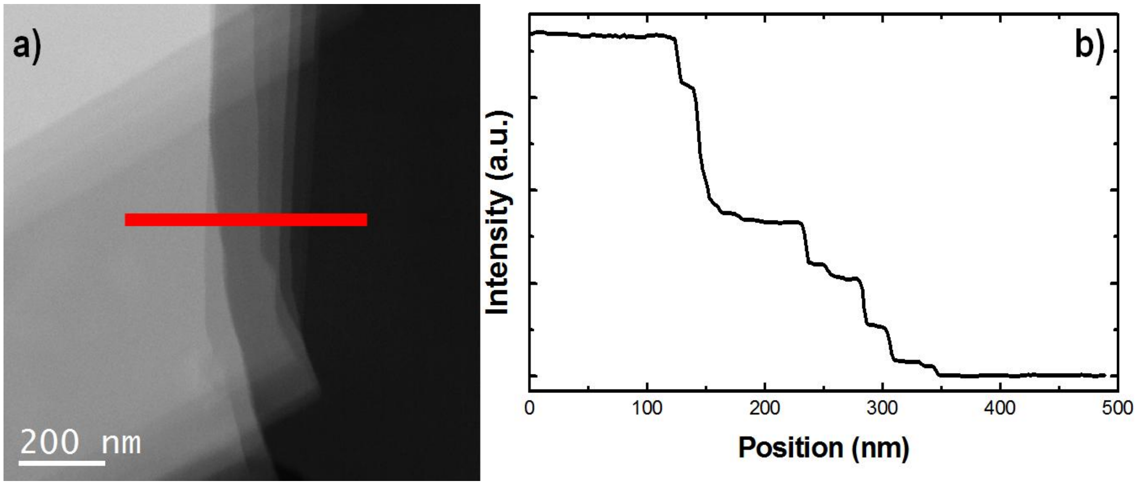

a) HAADF-STEM image of a flake edge. b) intensity line profile recorded on the red marked line showing that the flakes have stepped edges rather than sharp ones.

Supplementary Figure 7: Estimation of the number of layers composing the flake

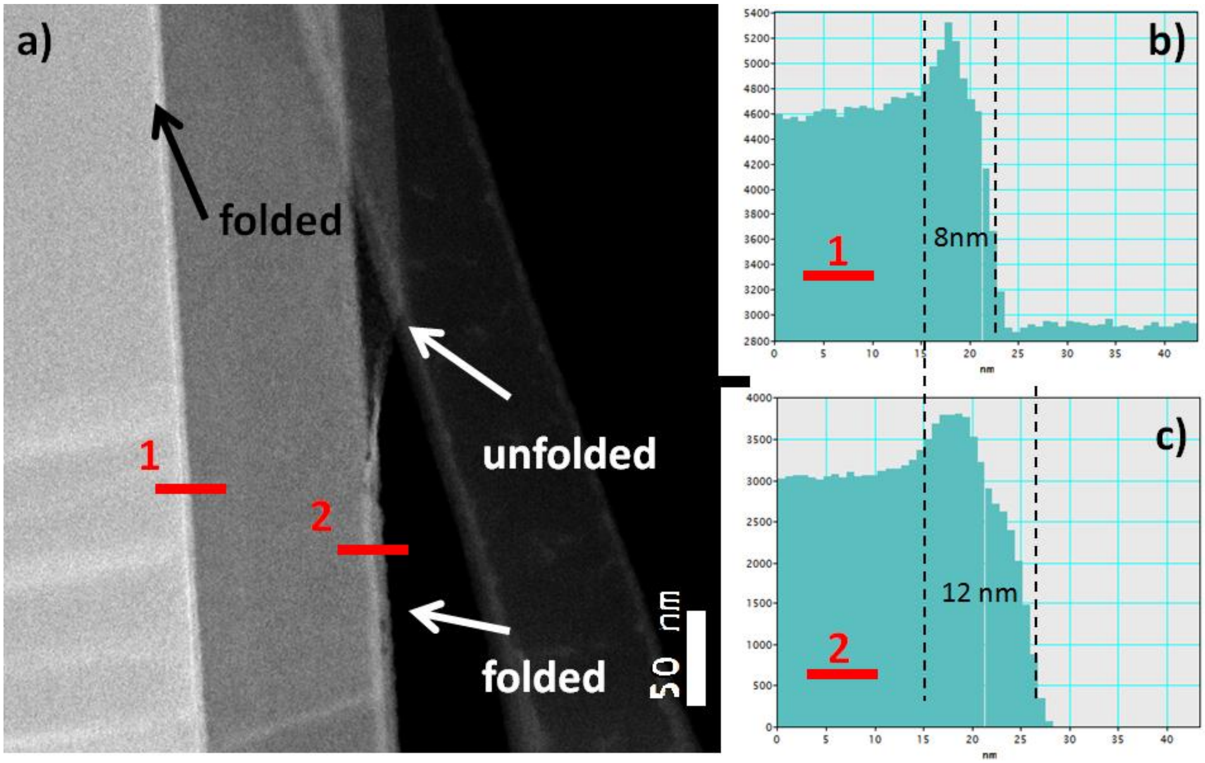

a) High magnification HAADF-STEM image of a flake edge. b) and c) intensity line profiles recorded along the red marked lines according to the labels.

Supplementary Figure 8: atomic resolution STEM-HAADF image of a MoS_2 nanoroll.

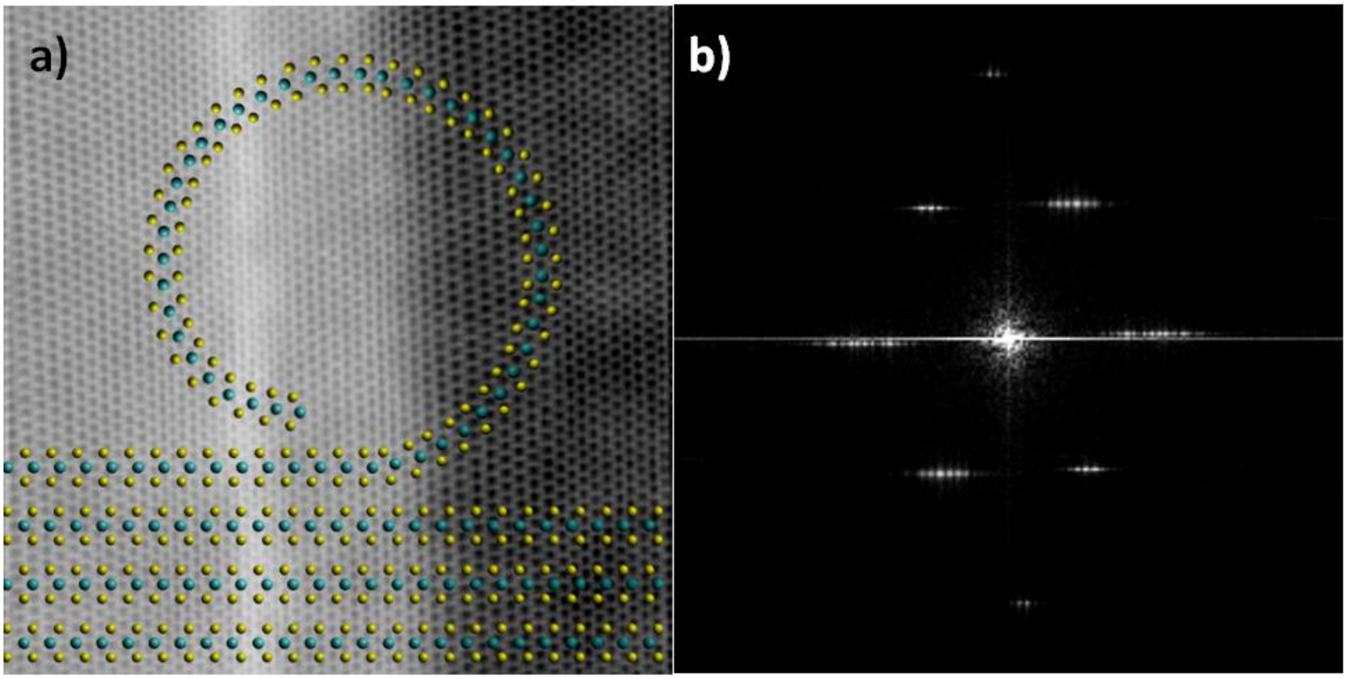

a) Atomic resolution HAADF-STEM image of a MoS_2 nanoroll and b) its fast Fourier transform.

Supplementary Figure 9: Energy dispersive X-ray microanalysis of pristine Molybdenite

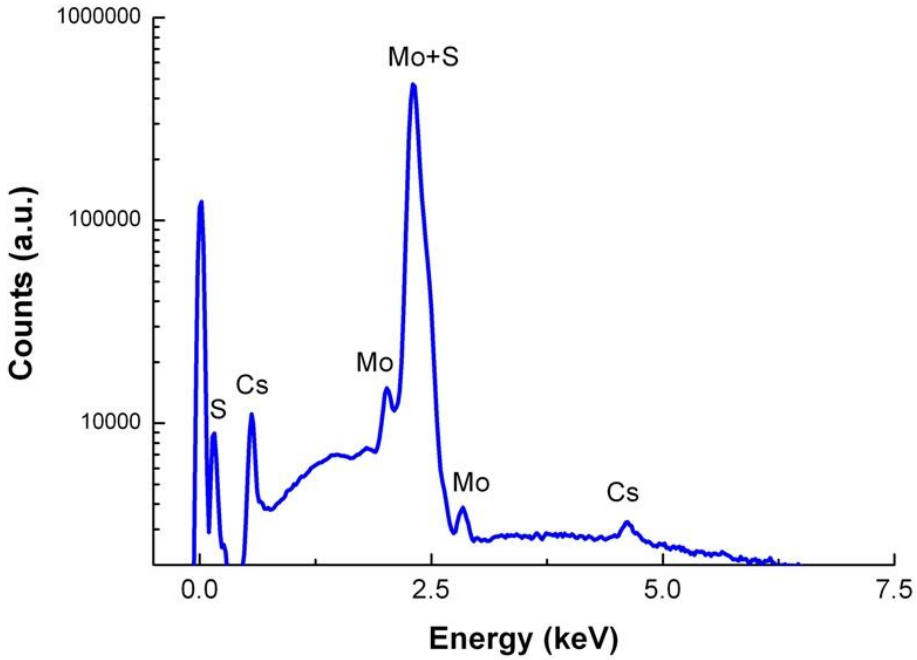

EDX quantitative analysis of geological Molybdenite.The exact stoichiometry is MoS_X, X=1.9. Cesium impurities with a concentration of about 1% [2] are also found.

Supplementary Figure 10: CL spectroscopy and imaging of an exfoliated flake

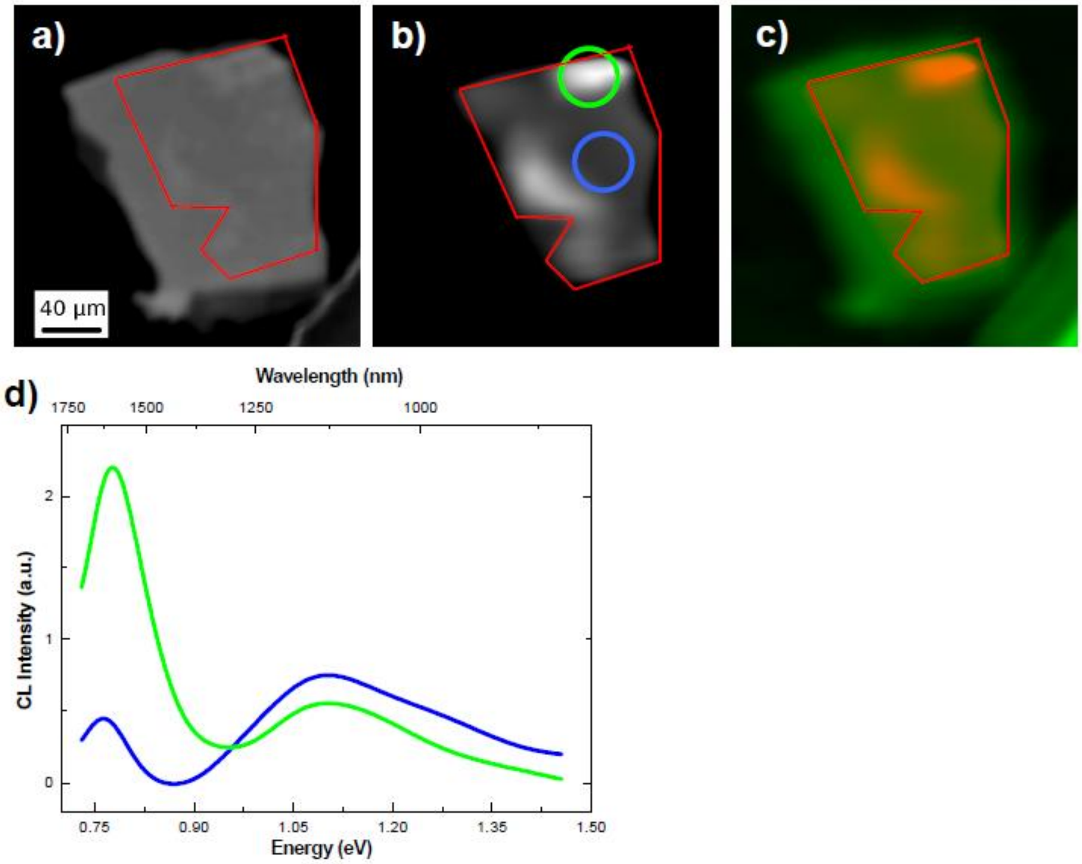

SE image of the multilayer MoS_2 flakes, b) 0.76 eV monochromatic CL map, c) superimposition of the SE (green scale) and CL signals (red scale). d) Spot mode CL spectra obtained in the areas highlighted by green and blue circles in b, the same color code is kept.

Supplementary Figure 11: High resolution X-Ray diffraction in in the symmetrical geometry

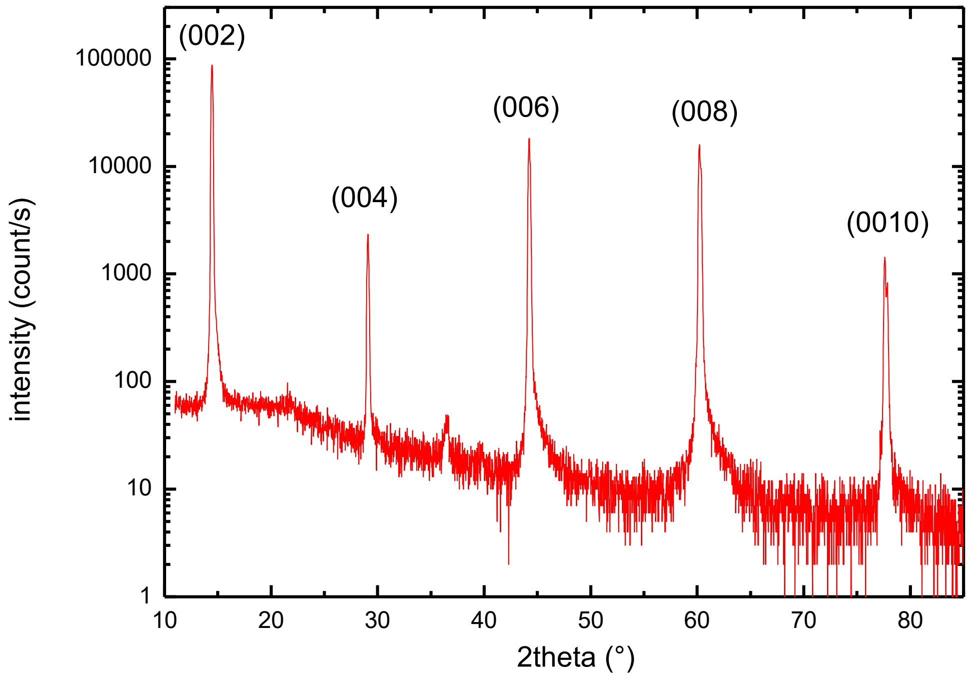

MoS_2 (00*l*) peaks are clearly shown and their position and intensity are compatible with that calculated for beta-MoS_2 reported in JCPDS-database [1].

Supplementary Figure 12: Zoom of the (0010) peak in Supplementary Figure 1

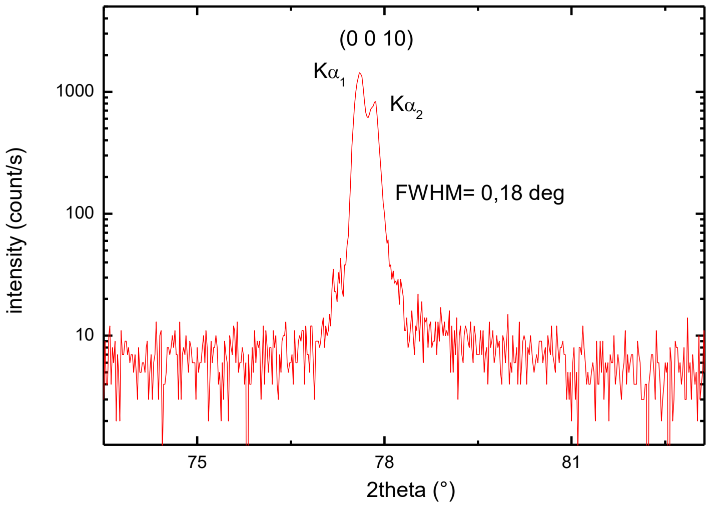

The splitting of the peak due to the $K\alpha_1$ and $K\alpha_2$ line of the source is reported

Supplementary Figure 13: X-ray diffraction of Molybdenite in asymmetric geometry

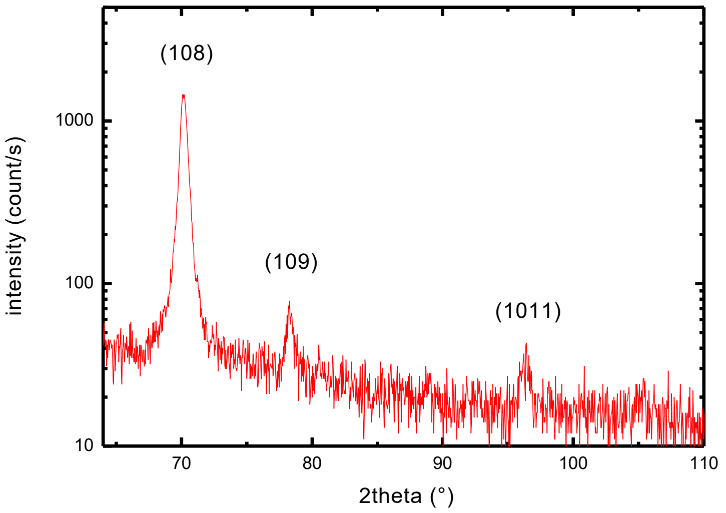

The position and the intensity of the peaks confirm the compatibility with the sample beta MoS_2. There is no evidence of mosaic spread.

Supplementary Figure 14: Raman mapping of E_{2g} and A_{1g} vibrational modes

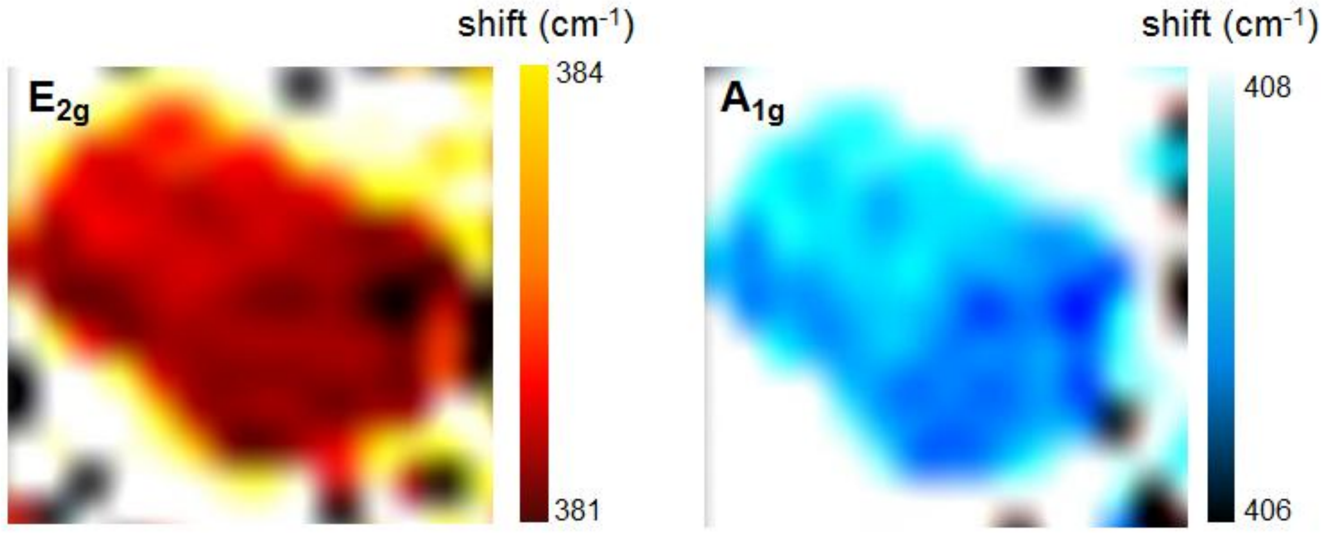

Raman map of the E_{2g} mode frequency position (left) and of the A_{1g} mode frequency position (right)

Supplementary Note 1 Additional Cathodoluminescence spectroscopy results

The Gaussian peak fitting in Supplementary Figure 2 reveals that slightly asymmetric peak at 1.25 eV is composed by two different components set at 1.29 eV and 1.23 eV. The peak at 1.29 eV is related to the band-to-band transition of the bulk MoS_2[1] . The 1.23 eV peak is probably due to shallow intra-bandgap levels related to some impurity as cesium [6] or rhenium [7]. The presence of these impurities is due to the geologic nature of molybdenite [8]. In addition the 1.25 eV peak shows a shoulder on the low energy side, reliable with a small components set at 1.14 eV.

The Gaussian peak fitting of Supplementary Figure 1 reveals that the strongly asymmetric peak at 1.07 eV is composed by three different components set at 1.25 eV, 1.10 eV and 0.98 eV. The peaks at 1.25 eV and 1.1 eV are reliable with two components previously found in the pristine molybdenite spectrum, meanwhile the 0.98 eV peak appears only after the mechanical exfoliation process and therefore we can suppose that it is related to the ripplocations formed during this process (see Supplementary Figure 33).

As for the luminescence yield of the 0.75-0.76 eV emission, since the detection system uses a lock in amplifier coupled to a Ge photodetector, it is extremely complicated to determine the quantum yield of a single emission. Therefore, to have an estimation of the efficiency of the 0.75-0.76 eV emission, we quantitatively compared the integrated intensities of the band at 1.25 eV from pristine molybdenite with the band at 0.75 eV from Vs in the flakes. The calculations are carried out on the single band obtained by Gaussian deconvolutions. In particular the integrated intensity of the 0.75-0.76 eV in the flakes spectrum is 0.09 a.u. meanwhile the value in the case of the band-to-band transition in the pristine MoS_2 spectrum is 0.06 a.u. (Supplementary Figure 3). As a result, the integrated intensity of the band peaked at 0.75 eV was about 30% larger than that of the band-to-band transition from pristine MoS_2.

The SE image of a MoS_2 multilayer flake, shown in Supplementary Figure 10, reveals a change in the SE contrast. This effect is probably related to a change in the flakes thickness, demonstrating the presence of areas composed by a different number of layers. The comparison of the SE image and the 0.76 eV monochromatic CL map suggests that the areas with a bright SE contrast and intense CL emission are thicker than the areas that do not present any CL intensity. The comparison of the CL spectra taken in area with different emission intensities obtained by the 0.76 eV CL monochromatic map (i.e. the center and the edge of the flakes) reveals that the 1.07 eV and 0.76 eV emissions result more intense in the center of the flakes and at the flake edge, respectively. It is worth noting that the 0.76 eV band appears also at the center of the flake, supporting the hypothesis that this emission is related to an intra-bandgap state. In addition the peak energy changes depending on the position of the spot mode CL spectra, the emission being peaked at 0.78 eV on the edge and at 0.76 eV in the center of the flake. This effect suggests that increasing the defect concentration the emission peak blue shifts.

Influence of electron beam irradiation on CL spectra

The effect of the electron beam exposure has been carefully considered and studied by CL spectroscopy. In order to understand the effect of the electron beam irradiation on the stability of the different CL emissions reported in our manuscript, we irradiated a MoS2 flake for 30 minutes in the SEM with the same parameters used for the CL characterization and then we acquired an additional CL spectrum (Supplementary Figure 4). The intensity of both the 0.76 eV and 1.12 eV emissions decrease following 30 min irradiation. In addition, the electron beam irradiation mainly affects the high energy tail of the 1.12 eV emission, due to the indirect band-to-band radiative transition of MoS_2. This effect is consistent with the formation of nonradiative centers due to the electron beam irradiation, as recently reported

in Ref 9, that affect the different emissions composing the CL spectrum.

Supplementary Note 2: Transmission Electron Microscopy analyses on the flakes edges

High Angle Annular Dark Field (HAADF-) STEM imaging is a technique highly sensible to thickness variations. The image reported in Supplementary Figure 6 has been acquired on the edge of a mechanically exfoliated flake. It reveals that the flaks have stepped edges rather than sharp ones. In fact, the different shades of gray correspond to step on the edge having different thicknesses and therefore exhibiting different contrast. This is well evident in the intensity line profile reported in Supplementary Figure 6. The line profile can be exploited to give rough estimation of the number of layer composing the flakes (at least 30 layers in the reported case).

The MoS_2 layers are usually terminated on the edge with an approximately 10 nm thick nanoroll, see profiles of Supplementary Figures 7b and 7c, due to the massive amount present there. The presence of the nanoroll is clearly demonstrated in Supplementary Figures 7a. Here, close to the flake edge, a C filament, from the TEM grid, is present. Thanks to electrostatic interaction between the flake and the C, the nanoroll unfolds. A clear signature of the nanoroll presence, is the brighter contrast, due to the locally increased thickness, visible in the line profile shown in Supplementary Figures 7c. The nanorolls are ubiquitously present where a MoS_2 layer is terminated as demonstrated by the line profile Supplementary Figures 11b measured from the thicker region of the flake.

In Supplementary Figure 8a we report an atomic resolution STEM-HAADF image of a MoS2 nanoroll. A schematic drawing of the flake cross section is superimposed to the image for a better interpretation of the image contrast which is proportional to the number of layers. In Supplementary Figure 8b the FFT of Supplementary Figure 12a is reported and it is consistent with the calculated diffraction expected for a MoS2 nanotube as reported by Seifert et al. [10]

In Supplementary Figure 5 is reported a low magnification STEM image of a typical flake showing the homogeneous distribution of ripplocations across the sample.

Supplementary Figure 14 reports on Raman maps, carried out in a z-backscattering geometry, of the E_{2g} and A_{1g} mode positioning used to identify the flake area in correspondence with the optical microscopy image in the Raman spectroscopy analysis.

Supplementary Note 3: Structural, compositional and optical characterization of Molybdenite

X-Ray diffraction analyses

Samples of geological MoS_2 were analyzed at room temperature and atmospheric pressure by X-ray diffraction. The in-plane θ-2θ scans are performed with a high resolution diffractometer with Goebel mirror selecting $K\alpha_1$, $K\alpha_2$ lines from a copper anode. In Supplementary Figure 11 is reported the symmetrical diffraction geometry. The surface of the sample is parallel to the basal plane of the hexagonal lattice cell with lattice parameters: *a*: 3.16116 Å *c*: 12.2985 Å The extra peaks between the (002) and (006) are due to multiple diffractions inside the sample. The zoom of the (00 10) reflection is reported in Supplementary Figure 12, in which the splitting of the peak due to the $K\alpha_1$ and $K\alpha_2$ line of the source is evident. The FWHM measured on (0010) peak is 0.09 deg on the omega scale, corresponding to the

instrumental broadening, equivalent to that of a perfect crystal. Supplementary Figure 13 reveals that there is no mosaic spread in the sample.

Energy Dispersive X-Ray Microanalysis of Molybdenite

The compositional analysis of Molybdenite is carried out by Energy Dispersed X-Ray spectroscopy (EDX) (Supplementary Figure 9). The analysis reveals that the geological molybdenite is naturally sulfur poor and the exact stoichiometry is MoS_X, X=1.9. In addition the EDX analysis reveals the presence of cesium impurities with a concentration of about 1% [2].

Cathodoluminescence spectroscopy

Supplementary Figure 3 shows the CL spectroscopic analysis of bulk molybenite. It is worth noting that geological molybdenite is used as reference for the light emission properties of bulk MoS_2. In addition we are able to compare the light emission of pristine (blue line) and cracked (red line) molybenite. The pristine molybdenite CL spectrum presents a peak at 1.25 eV, related to the indirect band-to-band transition of bulk MoS_2 [3]. As for the two emissions peaked at 1.14 eV and 1.05 eV, there is no clear attribution in the literature. We can however speculate that the two peaks could be related to intrinsic defects and/or surface states related emissions as for other metal sulfide materials [4,5]. In the case of the cracked molybdenite the 1.25 eV peak intensity decreases drastically with a concurrent broadening of the emission. A new intense emission at 0.79 eV appears in the cracked area.

Supplementary references

1. McMurdie, H. et al., Standard X-ray Diffraction Powder Patterns from the JCPDS Research Associateship, *Powder Diffraction*, 1 269 (1986)
2. A. Molle et al., Evidence of native Cs impurities and metal-insulator transition in MoS2 natural crystals, *Advanced Electronic Materials* March 2016
3. Mak, K. F., Lee, C., Hone, J., Shan, J. & Heinz, T. F. Atomically thin MoS 2: a new direct-gap semiconductor. *Physical Review Letters* **105**, 136805 (2010).
4. Wang, Z. L. *Nanowires and Nanobelts: Materials, Properties and Devices. Volume 1: Metal and Semiconductor Nanowires*. Vol. 1 (Springer Science & Business Media, 2013).
5. Sajeesh, T. H., Poornima, N., Kartha, C. S. & Vijayakumar, K. P. Unveiling the defect levels in SnS thin films for photovoltaic applications using photoluminescence technique. *physica status solidi (a)* **207**, 1934-1939, doi:10.1002/pssa.200925593 (2010).
6. Park, K. T., Richards-Babb, M., Hess, J. S., Weiss, J. & Klier, K. Valence-band electronic structure of ${¥mathrm{MoS}}_{2}$ and Cs/MoS_2 studied by angle-resolved x-ray photoemission spectroscopy. *Physical Review B* **54**, 5471-5479 (1996).
7. Tiong, K. K., Liao, P. C., Ho, C. H. & Huang, Y. S. Growth and characterization of rhenium-doped MoS2 single crystals. *Journal of Crystal Growth* **205**, 543-547, (1999).

8. Addou, R., Colombo, L. & Wallace, R. M. Surface Defects on Natural MoS2. *ACS Applied Materials & Interfaces* **7**, 11921-11929, (2015).
9. Rotunno, E., Fabbri, F., Cinquanta, E., Kaplan, D., Longo, M., Lazzarini, L., Molle, A., Swaminathan, V., and Salviati, G., Structural, optical and compositional stability of MoS2 multi-layer flakes under high dose electron beam irradiation *2D Materials* **3**, 025024 (2016).
10. Seifert, G., Terrones, H., Terrones, M., Jungnickel, G., and Frauenheim, T., Structure and electronic properties of MoS_2 nanotubes *Physical review letters* **85**, 146 (2000).
